# Complications of continuous renal replacement therapy in critically ill children: a prospective observational evaluation study

**DOI:** 10.1186/cc8172

**Published:** 2009-11-23

**Authors:** Maria J Santiago, Jesús López-Herce, Javier Urbano, María José Solana, Jimena del Castillo, Yolanda Ballestero, Marta Botrán, Jose María Bellón

**Affiliations:** 1 Pediatric Intensive Care Service, Hospital General Universitario Gregorio Marañón, Dr Castelo 47 Madrid, 28009, Spain; 2 Preventive and Quality Control Service, Hospital General Universitario Gregorio Marañón, Dr Castelo 47 Madrid, 28009 Spain

## Abstract

**Introduction:**

Continuous renal replacement therapy (CRRT) frequently gives rise to complications in critically ill children. However, no studies have analyzed these complications prospectively. The purpose of this study was to analyze the complications of CRRT in children and to study the associated risk factors.

**Methods:**

A prospective, single-centre, observational study was performed in all critically ill children treated using CRRT in order to determine the incidence of complications related to the technique (problems of catheterization, hypotension at the time of connection to the CRRT, hemorrhage, electrolyte disturbances) and their relationship with patient characteristics, clinical severity, need for vasoactive drugs and mechanical ventilation, and the characteristics of the filtration techniques.

**Results:**

Of 174 children treated with CRRT, 13 (7.4%) presented problems of venous catheterization; this complication was significantly more common in children under 12 months of age and in those weighing less than 10 kg. Hypotension on connection to CRRT was detected in 53 patients (30.4%). Hypotension was not associated with any patient or CRRT characteristics. Clinically significant hemorrhage occurred in 18 patients (10.3%); this complication was not related to any of the variables studied. The sodium, chloride, and phosphate levels fell during the first 72 hours of CRRT; the changes in electrolyte levels during the course of treatment were not found to be related to any of the variables analyzed, nor were they associated with mortality.

**Conclusions:**

CRRT-related complications are common in children and some are potentially serious. The most common are hypotension at the time of connection and electrolyte disturbances. Strict control and continuous monitoring of the technique are therefore necessary in children on CRRT.

## Introduction

Continuous renal replacement therapy (CRRT) is currently the most widely used technique for extrarenal filtration in critically ill children, because it allows continuous and programmed fluid removal [1-5].

Although a number of studies have demonstrated that these techniques are useful and safe in critically ill children of any age [4-7], complications do occur [8]. Children are at a higher risk than adults for developing complications associated with CRRT due to the difficulty of venous catheterization with the large-caliber catheters required for the technique, the large extracorporeal volume of the system (filters and lines), which predisposes to hypotension at the time of connection, and the need for a more accurate control of volumes in order to avoid fluid and electrolyte disturbances.

There are no studies that have prospectively analyzed the complications or risk factors in children on CRRT.

The objective of the present study was to determine the incidence of complications in children requiring CRRT and to analyze the predisposing risk factors.

## Materials and methods

An analysis was performed of the data from a prospective, single-center register of critically ill children treated using CRRT. The study was approved by the local Institutional Review Board and due to the characteristics of the study inform consent of patients was not considered to be necessary. Between January 1996 and June 2009, CRRT techniques were used in 174 children (105 boys (60.3%) and 69 girls (39.7%)) with a mean (standard deviation) age of 52.3 (63.8) months and weight of 17.6 (18.2) kg; 43.7% of the patients were under one year of age. The most common conditions in patients requiring CRRT were heart disease (55.7%), particularly during the postoperative period of cardiac surgery, and sepsis (19.5%).

Two different renal replacement pumps were used to perform CRRT: the BSM321C (Hospal^®^, Barcelona, Spain) in the first 35 patients and the Prisma (Hospal^®^, Barcelona, Spain) in the remaining 139. The caliber of the catheters used was between 4F and 11F and the filters were between 0.04 m^2 ^and 0.9 m^2^, according to the age and weight of the patient. All patients received continuous anticoagulation with heparin to maintain an activated coagulation time between 130 and 200 seconds. Other anticoagulant or antiaggregant drugs (citrate, warfarin, aspirin, prostacyclin) were not administered.

The following data were gathered prospectively in all patients on starting CRRT: age; weight; sex; diagnosis; severity scores, pediatric risk of mortality (PRISM II) score [9], pediatric index of mortality (PIM I and II) score [10], pediatric logistic organ dysfunction (PELOD) score only from 2001 [11]; number of organ failures; blood pressure; need for vasoactive drugs; dose of dopamine and adrenaline; lactic acid levels; pH and base excess; levels of creatinine, urea, alanine transaminase (ALT), bilirubin, sodium, potassium, chloride, calcium, phosphorus, magnesium, albumin and platelets; and type of filtration pump used. The type of connection to CRRT was determined by the physician responsible for the patient. In some cases the connection was made directly to the circuit that had previously been primed using normal saline, in others, after purging with heparin, the system was flushed using 5% albumin before connection to the patient. During filtration, a daily record was kept of the technique used (hemodiafiltration or hemofiltration), maximum dose of heparin, ultrafiltration rate, life of each filter, electrolyte levels, complications related to the CRRT, and mortality during admission to the pediatric intensive care unit.

The following complications were analyzed: 1) complications of catheterization, defined as hemorrhage with a fall of more than 2 g/dL in the hemoglobin concentration and/or hypotension or the need for transfusion and/or withdrawal of the catheter from that site, thrombosis, pneumothorax, and altered limb perfusion; 2) hypotension on connection to the filter, defined as a fall in the mean blood pressure (MBP) of more than 20 mmHg over baseline and/or an MBP more than two standard deviations below the normal values for age and that required volume expansion and/or an increase in the dose of vasoactive drugs that the patient was receiving in the first 60 minutes after the connection to CRRT; 3) significant hemorrhage, defined as a fall of more than 2 g/dL in the hemoglobin concentration in the first 24 hours after bleeding and/or hypotension and that required packed red cell transfusion; and 4) electrolyte disturbances including hyponatremia (sodium <130 mEq/L), hypernatremia (sodium >150 mEq/L), hypokalemia (potassium <3 mEq/L), hyperkalemia (potassium >5.5 mEq/L), hypochloremia (chlorine <95 mEq/L), hyperchloremia (chlorine >115 mEq/L), hypocalcemia (total calcium <8 mg/dL), hypercalcemia (total calcium >12 mg/dL), hypophosphatemia (phosphate <4 mg/dL in children <6 years and phosphate <3 mg/dL in children >6 years), hyperphosphatemia (phosphate >7 mg/dL), hypomagnesemia (magnesium <1.5 mg/dL), and hypermagnesemia (magnesium >3 mg/dL). An analysis was performed of the changes in the electrolyte levels during the first three days of CRRT. The incidence of complications of CRRT between the first seven years and the second seven years of the study was compared.

The statistical analysis of the results was performed using the SPSS statistical package version 14.0. Pearson's chi-squared test and Fisher's exact test were used to compare percentages and the Mann-Whitney test to compare values with a non-parametric distribution. Significance was taken as a *P *value less than 0.05.

## Results

### Complications of catheterization

Complications of catheterization for CRRT occurred in 13 patients (7.4%), four of whom presented more than one such complication. The complications included hematoma at the puncture site (6 cases, 3.4%), hemorrhage (4 cases, 2.2%), altered venous drainage of the lower limbs (6 cases, 3.4%), and incorrect position of the jugular venous catheter requiring change (1 case, 0.05%). There were no cases of pneumothorax or hemothorax. Patients with complications of catheterization had a significantly lower age and weight than the other patients, and these complications were most common in children younger than 12 months of age and with a weight of less than 10 kg (Table 1).

**Table 1:** Risk factors of catheterization complications in children with CRRT

	With complications		Without complications		*P*
	Mean	SD	Mean	SD	
**Age **(months)	20.2	39.4	53.6	64.2	0.016

**Weight **(kg)	8.0	10.4	17.9	18.4	0.014

**PRISM score**	14.7	8.4	14.7	8.4	0.596

**PIM score**	-2.8	1.4	-1.9	1.4	0.181

**PELOD score**	23.2	3.9	17.2	8.2	0.095

**Number of failed organs**	2.8	1.0	2.9	1.1	0.922

**Lactic acid **(mmol/L)	2.1	1.2	3.1	3.7	0.725

**Arterial pH**	7.27	0.1	7.32	0.1	0.416

**Mean blood pressure **(mmHg)	53.7	27	62.0	17.9	0.173

**Dose of adrenaline **(μg/kg/min)	0.3	0.2	0.5	1.2	0.914

**Dose of dopamine **(μg/kg/min)	8.4	9.5	9.1	6.3	0.208

**Initial creatinine **(mg/dL)	1.3	1	1.5	1.4	0.825

**Initial urea **(mg/dL)	92.0	83.1	83.4	57.9	0.835

**ALT **(UI/L)	89.7	151.3	222.3	756.6	0.977

**Billirubin **(mg/dL)	1.5	1.5	1.9	2.3	0.781

**Initial platelet count**	158.583	124.291	175.425	188.538	0.821

	**Complications Number %**		**Complications Number %**		

**Age**	**<12 m**		**>12 m**		

	10	13.2	3	3.1	0.013

**Sex**	**Male**		**female**		

	8	7.8	5	7.2	0.889

**Weight**	**<10 kg**		**>10 kg**		

	11	12.2	2	2.4	0.015

**Diagnoses**	**Cardiopathies**		**Rest of diagnoses**		

	5	5.3	8	10.4	0.206

**Mechanical ventilation**	**Yes**		**No**		

	11	7.6	2	7.4	0.974

**Vasoactive drugs**	**Yes**		**No**		

	10	7.4	3	8.3	0.843

**Initial MAP**	**<55 mmHg**		**>55 mmHg**		

	8	11.4	5	4.4	0.112

**Initial hypotension**	**Yes**		**No**		

	7	8.3	6	6	0.530

**Dose of adrenaline**	**> 0.6 μg/kg/min**		**> 0.6 μg/kg/min**		

	3	8.8	7	6.4	0.632

**Catheter size**	**4 to 6.5 Fr**		**>6.5 Fr**		

	10	9.8	2	3.8	0.183

**Mortality**	**Yes**		**No**		

	4	30.8	9	36.5	0.680

ALT = alanine transferase; CRRT = continuous renal replacement therapy; MAP = mean arterial pressure; PELOD = pediatric logistic organ dysfunction; PIM = pediatric index of mortality; PRIMS = pediatric risk of mortality; SD = standard deviation.

There was no relationship between the complications of catheterization and the diagnosis, clinical severity of the patients at the time of starting the technique, need for mechanical ventilation, caliber of the catheter, initial platelet count (Table 1), or venous access used (subclavian, 5%; jugular, 7.7%; femoral, 8.2%; *P *= 0.912).

The incidence of complications of catheterization was higher in the first period of the study 14.5% than in the second period (4.2%; *P *= 0.01).

None of the complications of catheterization gave rise to serious clinical repercussions or prevented the use of CRRT. There was no relation between the complications of catheterization and mortality.

### Hypotension at the time of connection to the CRRT

Before connecting to CRRT, 72 patients (41.3%) had hypotension. Hypotension was more common in children with heart disease, with greater clinical severity at the time of starting CRRT (evaluated using the PRISM, PIM, and PELOD scores, number of organ failures, lactic acid levels, MBP, need for mechanical ventilation or vasoactive drugs, dose of adrenaline and dopamine before starting CRRT, and liver function (ALT and bilirubin). Children with previous hypotension had a significantly higher mortality than the other children.

On the other hand, hypotension soon after connecting the CRRT occurred in 53 patients (30.4%). Hypotension on connection to the CRRT was not statistically associated with any patient or CRRT characteristics and there were no differences in the incidence of hypotension between the two periods of the study (Table 2).

**Table 2:** Risk factors of hypotension during connection of CRRT in children

	Hypotension		No hypotension		*P*
	Mean	SD	Mean	SD	
**Age **(months)	53.4	67.5	50.0	61.4	0.694

**Weight **(kg)	18.1	19.8	16.9	17.3	0.910

**PRISM score**	16.8	15.5	21.6	25.8	0.650

**PIM score**	10.1	13.4	10.9	16.8	0.874

**PELOD score**	25.7	30.2	20.7	22.7	0.294

**Number of failed organs**	3.2	1.2	2.8	1.1	0.139

**Lactic acid **(mmol/L)	3.2	3.3	3.0	3.8	0.357

**Arterial pH**	7.32	0.12	7.31	0.10	0.341

**MAP **(mmHg)	58.2	14.8	62.9	20.2	0.124

**Dose of adrenaline **(μg/kg/min)	0.4	0.5	0.57	1.3	0.734

**Dose of dopamine **(μg/kg/min)	9.1	6.6	9.0	6.6	0.932

**Initial creatinine **(mg/dL)	1.4	1.1	1.5	1.3	0.491

**Initial urea **(mg/dL)	79.2	61.4	86.2	59.6	0.236

**ALT **(UI/L)	523.5	1261.7	71.0	112.5	0.212

**Billirubine **(mg/dL)	1.8	1.6	1.8	2.4	0.667

**Extracorporeal circuit volume/weight of patient **(ml/kg)	8.6	4.6	8.8	5.3	0.982

	**Hypotension** **Number %**		**Hypotension** **Number %**		

**Age**	**<12 m**		**>12 m**		

	24	31.6	29	30.2	0.847

**Sex**	**Male**		**Female**		

	34	33	19	27.5	0.446

**Weight**	**<10 kg**		**>10 kg**		

	27	30	35	31.7	0.809

**Diagnoses**	**Cardiopathies**		**Rest of diagnoses**		

	31	32.6	22	28.6	0.566

**Mechanical ventilation**	**Yes**		**No**		

	48	33.1	5	18.5	0.132

**Vasoactive drugs**	**Yes**		**No**		

	42	30.9	11	30.6	0.970

**Initial MAP**	**<55 mmHg**		**>55 mmHg**		

	24	38.1	25	27.5	0.164

**Dose of adrenaline**	**> 0.6 μg/kg/min**		**> 0.6 μg/kg/min**		

	12	35.3	32	29.4	0.513

**Filter surface**	**<0.3 m^2^**		**>0.3 m^2^**		

	21	33.3	32	24.4	0.586

**Extracorporeal circuit volume/weight of patient**	**> 5 ml/kg**		**< 5 ml/kg**		

	36	32.1	10	22.2	0.217

**Mortalilty**	**Yes**		**No**		

	24	38.7	29	26.4	0.095

ALT = alanine transferase; CRRT = continuous renal replacement therapy; MAP = mean arterial pressure; PELOD = pediatric logistic organ dysfunction; PIM = pediatric index of mortality; PRIMS = pediatric risk of mortality; SD = standard deviation.

It was not possible to determine whether priming with albumin was associated with a need for lower volume expansion or less increase in the dose of vasoactive drugs. We only recorded if hypotension developed and whether or not volume expansion or an increase in the dose of drugs was required, not the actual volume of fluids or dose of drugs administered.

### Hemorrhage

Clinically significant hemorrhage during CRRT occurred in 18 patients (10.3%). There was no relation between the presence of hemorrhage and age, weight, diagnosis, or clinical severity at the start of CRRT (Table 3). Although the platelet counts were slightly lower in children with hemorrhage, the differences did not reach statistical significance at any time during the course of treatment (Table 3). However, patients with bleeding did receive platelet transfusions more frequently. The maximum doses of heparin administered did not differ significantly between patients with hemorrhage and other patients (Table 3). Patients with hemorrhage presented a higher mortality than other patients, although the differences did not reach statistical significance (*P *= 0.068; Table 3).

**Table 3:** Risk factors of bleeding complications in children with CRRT

	Bleeding		No bleeding		*P*
	Mean	SD	Mean	SD	
**Age **(months)	64.2	70.8	49.5	62.3	0.385

**Weight **(kg)	22.7	22.0	16.6	17.5	0.310

**PRISM score**	15.0	7.0	14.7	8.5	0.643

**PIM score**	-2.1	0.9	-2.0	1.5	0.929

**PELOD score**	18.1	9.2	17.5	8.1	0.402

**Number of failed organs**	3.3	1.1	2.9	1.1	0.189

**Lactic acid **(mmol/L)	4.3	5.4	2.9	3.4	0.530

**Arterial pH**	7.31	0.1	7.31	0.1	0.906

**MAP **(mmHg)	56.2	22.7	62.0	18.2	0.211

**Dose of adrenaline **(μg/kg/min)	0.5	0.6	0.5	1.2	0.409

**Dose of dopamine **(μg/kg/min)	10.9	8.2	8.8	6.3	0.295

**Initial creatinine **(mg/dL)	1.2	0.8	1.5	1.4	0.956

**Initial urea **(mg/dL)	93.8	68.9	82.9	59.0	0.475

**ALT **(UI/L)	174.7	171.0	209.5	776.3	0.355

**Billirubine **(mg/dL)	3.1	4.0	1.5	1.4	0.218

**Initial platelet **(inicial)	150.944	119.341	177.289	170.637	0.821

**Platelet after 24 hours of CRRT**	89.000	72.960	122.068	111.964	0.254

**Platelet after 48 hours of CRRT**	93.000	76.183	105.589	99.070	0.657

**Maximum dose of heparin **(UI/kg/h)	14.7	13.1	15.2	10.3	0.351

**Ultrafiltration rate **(mL/kg/h)	43.6	34.4	37.3	25.3	0.751

**Circuit lifespan **(h)	47.5	61.5	41.9	36.9	0.638

	**Bleeding Number %**		**Bleeding Number %**		

**Age**	**<12 m**		**>12 m**		

	9	11.8	9	9.4	0.600

**Sex**	**Male**		**Female**		

	9	8.7	9	13	0.366

**Weight**	**<10 kg**		**>10 kg**		

	9	10	9	11	0.835

**Diagnoses**	**Cardiopathies**		**Rest of diagnoses**		

	10	10.5	8	10.4	0.977

**Mechanical ventilation**	**Yes**		**No**		

	17	11.7	1	3.7	0.211

**Vasoactive drugs**	**Yes**		**No**		

	15	11	3	8.3	0.638

**Initial MAP**	**< 55 mmHg**		**> 55 mmHg**		

	11	17.5	7	6.6	0.034

**Initial hypotension**	**Sí**		**No**		

	10	14.3	8	8.3	0.241

**Dose of adrenaline**	**>0.6 μg/kg/min**		**>0.6 μg/kg/min**		

	5	14.7	13	11	0.561

**Platelet**	**<50.000**		**>50.000**		

	6	20	12	10.3	0.152

**Platelet transfusion**	**Sí**		**No**		

	14	18.9	4	4.1	0.002

**CRRT technique**	**Hemofiltration**		**Hemodiafiltration**		

	3	6.7	15	11.8	0.333

**Filter surface**	**<0.3 m2**		**>0.3 m2**		

	9	14.3	9	14.7	0.937

**Maximum dose of heparin**	**>10 U/kg/h**		**<10 U/kg/h**		

	7	8.4	11	12.4	0.401

**Mortality**	**Yes**		**No**		

	10	55.6%	8	33.8%	0.068

ALT = alanine transferase; CRRT = continuous renal replacement therapy; MAP = mean arterial pressure; PELOD = pediatric logistic organ dysfunction; PIM = pediatric index of mortality; PRIMS = pediatric risk of mortality; SD = standard deviation.

The incidence of clinically significant hemorrhage was slightly higher in the first period of the study (14.5%) than in the second period (8.5%), however, the difference was not significant (*P *= 0.2).

### Electrolyte disturbances

**Figure 1:**
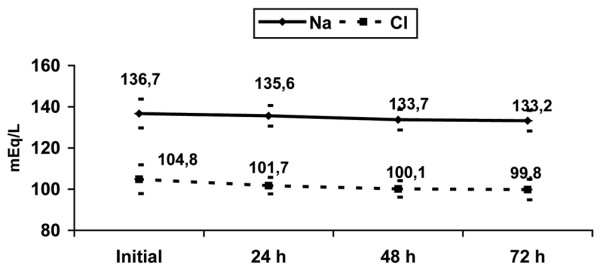
Evolution of sodium and chloride serum levels during the first 72 hours of continuous renal replacement therapy. Mean and standard deviation.

**Figure 2:**
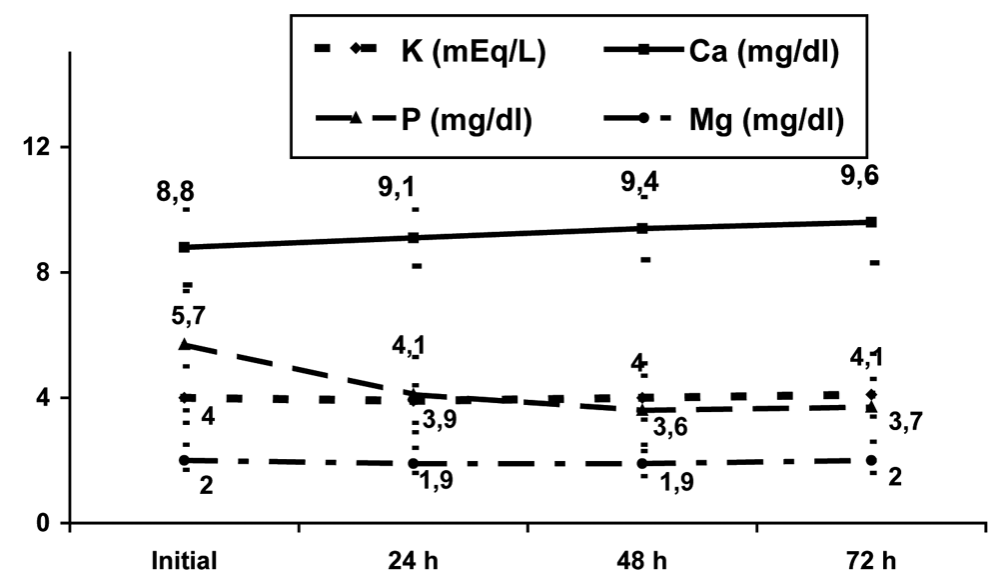
Evolution of potassium, calcium, phosphorus and magnesium serum levels during the first 72 hours of continuous renal replacement therapy. Mean and standard deviation.

**Figure 3:**
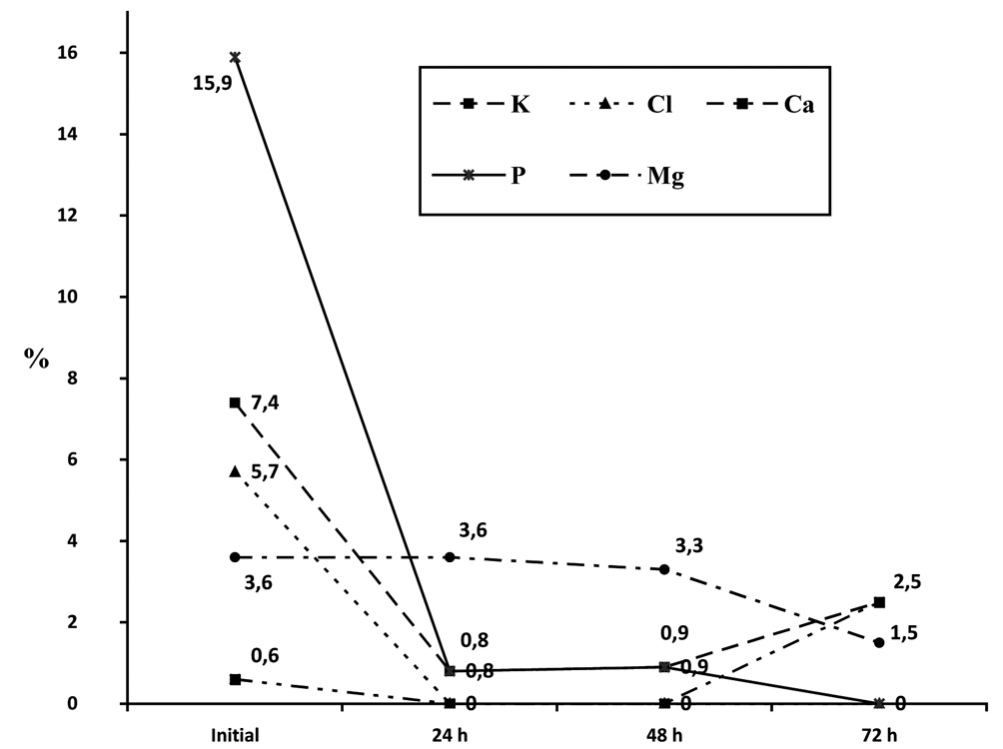
Percentage of patients with high serum levels of electrolytes during the first 72 hours of continuous renal replacement therapy. Mean and standard deviation.

**Figure 4:**
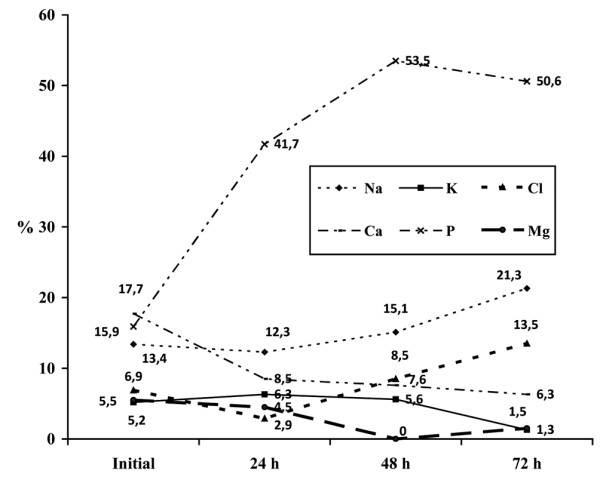
Percentage of patients with low serum levels of electrolytes during the first 72 hours of continuous renal replacement therapy. Mean and standard deviation.

The changes in the electrolyte levels (sodium, potassium, chloride, calcium, phosphorus, and magnesium) over the first 72 hours of CRRT are shown in Figures 1 and 2. In the first 72 hours of CRRT, the levels of sodium, chloride, and phosphate fell significantly, total calcium increased significantly, and the levels of potassium and magnesium remained unaltered. Figures 3 and 4 show the percentage of electrolyte disturbances during the first three days of CRRT. The percentage of patients with raised electrolyte levels decreased progressively during the first three days of therapy (Figure 3). In contrast, the percentage of patients with hyponatremia, hypochloremia, and hypophosphatemia increased significantly during CRRT, requiring an increase in the concentration of these electrolytes in the dialysis and replacement fluids and/or intravenous supplements (Figure 4). The electrolyte disturbances did not lead to clinical manifestations except in one patient in whom Dialisan AFB (Hospal^®^, Barcelona, Spain) was used as the dialysis fluid. This fluid has a sodium concentration of 4725 mEq/L and requires dilution of 1 mL in 35 mL of water before use; in error, the solution was used undiluted for a few hours and the patient presented hypernatremia of 216 mEq/L, hyperchloremia of 189 mEq/L, and ionic calcium of 4 mmol/L, leading to hypertension and a convulsive crisis. The electrolyte disturbances were corrected by substitution of the dialysis fluid (by a specific CRRT dialysis fluid) [12]; after correction, the patient presented a good clinical course and there have been no neurological or renal sequelae after nine years of follow-up.

The alterations in the electrolyte levels during the course of the study were not related to any of the variables analyzed or to the filtration technique used. There was no correlation between mortality and any of the electrolyte disturbances during the course of the study (data not shown).

## Discussion

Our study is the first that has prospectively investigated complications related to CRRT in critically ill children and that has analyzed the factors associated with these complications.

The percentage of complications of venous catheterization was similar to that found in other studies of central line catheterization in pediatric patients, despite the fact that the catheters necessary for CRRT are larger [13-15]. The complications of catheterization were more common in smaller children because catheterization is more difficult in these patients and because the caliber of the catheter used in infants is proportionally larger than in older children. In contrast to other series, we did not find differences in the rate of complications between the use of veins in the upper body (jugular and subclavian) or lower body (femoral) [13,15]. A recent study in adults that compared jugular and femoral venous access for acute CRRT found that the incidence of hematomas was higher in jugular than in femoral access, with no significant differences in the rates of infection secondary to catheterization [16]. The incidence of catheter-related infection was not analyzed in the present study. A recent study has shown that ultrasound-guided central venous catheter placement decreases the complications of catheterization, although we have not used this method in our patients [17].

Hypotension after connection to the CRRT system was the most common complication; it is more common in children because the extracorporeal volume of the circuit and filter used for CRRT represents 10 to 5% of a patient's blood volume [5]. The circuits used in our study have a priming volume (including the filter) of 50, 100, and 130 mL depending on the surface area of the filter used (0.04, 0.6, or 0.9 m^2^, respectively). Although the circuit priming volume is proportionally larger in children of lower weight, we did not find any relation between the frequency of hypotension and age, weight, or surface area of the filters. The design of filters and circuits with a low priming volume is an essential factor in the reduction of hemodynamic complications at the time of connection to the system.

Patients with previous hemodynamic alterations theoretically could have more hypotension after connection to the CRRT. However, surprisingly in our study, we have not found any individual factors associated with hypotension after CRRT connection. It is possible that, although individually each risk factor is not associated with hypotension, the combination of several risk factors such as the extracorporeal volume of the circuit and filter and the previous hemodynamic alterations could influence the development of hypotension after connection to CRRT.

There are a number of techniques that can be used to attempt to reduce the risk of hypotension at the time of connection, such as priming the circuit with whole blood or colloids, although there are no studies that have analyzed their efficacy. Patients on CRRT usually received many blood transfusions. To reduce the risks of transfusion, we decided to more frequently prime the circuit with 5% albumin rather than with whole blood if the hemoglobin is not very low. However, in our study, we did not record in which children the circuit was primed with albumin and we cannot therefore analyze the efficacy of this measure. Further studies are necessary to determine the efficacy of circuit-priming methods in the reduction of hypotension at the time of connection.

The treatment of hypotension was different depending on the situation of each patient. Generally, we used volume expansion with colloids 10 to 20 ml/kg as the first measure. If the hypotension was severe we also increased the vasoactive drugs that the patient received, and when the haemoglobin level was low we also transfused packed red cells.

Some authors have reported the onset of a bradykinin release syndrome when using filters with the AN69 membrane primed with blood; the syndrome presents as acute hypotension and can be avoided by raising the pH [18]. Although we use AN69 membranes, we have not had this complication because we do not prime the circuit with blood.

Hypotension can also occur if excessive ultrafiltration is programmed [5] or if the machine systems that measure the volumes function incorrectly. To prevent this, both the fluid balances measured by the CRRT machine and the clinical state of the patient should be monitored continuously. According to our protocol, nurses measured hourly the input and output fluid balance and checked the ultrafiltrate volume registered by the machine. Furthermore a continuous clinical vigilance was performed. According to these data the programming of the ultrafiltration was changed by the intensivist. We think that for this reason we have not found any complications of excessive ultrafiltration in our patients.

The need for anticoagulation of the CRRT system, associated with the frequent alterations of coagulation that occur in these patients, increases the risk of hemorrhage. Both CRRT and heparin can produce a fall in the platelet count, as found in our study, or an alteration of platelet function. Heparin continues to be the most widely used method of anticoagulation in CRRT [19], although it has been suggested that anticoagulation using sodium citrate could reduce the risk of heparin-related hemorrhage; however, sodium citrate increases the risk of hypocalcemia and alkalosis [5,20].

Although premature coagulation of the CRRT filter is more common in children [21], 10% of our patients presented clinically significant hemorrhage, and there was a higher mortality among these patients. In our study, we found no relationship between the incidence of hemorrhage and the platelet counts or doses of heparin used. However, an important limitation in our study is that no analysis was performed of a possible relationship between hemorrhage and other disturbances of coagulation. Moreover, it is also possible that patients with high risk of haemorrhage received a low dose of heparin and this fact could influence to not find relationship between heparin dose and bleeding. Hemorrhage in critically ill patients on CRRT is probably the consequence of several factors: a coagulation disorder, altered tissue perfusion caused by the underlying disease, and the alterations of coagulation caused by the extracorporeal circuit and anticoagulation [8,22].

Electrolyte disturbances are very common in critically ill children [23]. CRRT can be used to correct severe electrolyte disturbances, but can also produce them [24]. The risk is higher if inappropriate dialysis and/or replacement fluids are used [25], as occurred in one of our patients [12]. In our study, despite using balanced solutions, there was a significant fall in the levels of sodium, chloride, and phosphate, leading to the need to increase the concentration of these electrolytes in the dialysis and replacement fluids or to administer intravenous supplements. When balanced solutions are used high dose of dialysis and/or replacement fluids should not produce more electrolytes disorders. However we have not analyzed if electrolytes disorders were associated to the intensity of fluid dose prescriptions. Hyponatremia may develop if the dialysis and replacement fluids do not compensate the negative sodium balance [8]. In a previous study we found a very high incidence of hypophosphatemia in children on CRRT; this was due to the high efficacy of these techniques and the fact that the usual replacement and dialysis fluids do not contain phosphate [26]. The addition of phosphates to replacement and dialysis fluids did not cause any instability of the solutions or other complications, and reduced the incidence of hypophosphatemia and the need for intravenous phosphate supplements [26]. Therefore, as electrolyte disturbances are common in children on CRRT, periodic controls of their blood levels should be performed and the concentration in the replacement and dialysis fluids should be monitored closely in order to detect errors in the preparation of the fluids.

Other complications have been reported in patients on CRRT, such as alkalosis secondary to the bicarbonate content of the dialysis and replacement fluids [27], and errors of drug dose [28]. These complications were not analyzed in our study. Another limitation of our study is that we did not determine the incidence of hypothermia, which is more common in children on CRRT due to extracorporeal radiant heat exchange, or catheter-related infection [8].

## Conclusions

We conclude that the frequency of complications in children on CRRT is high, and some of these complications can be serious. The most common are hypotension at the time of connection and electrolyte disturbances. The hemodynamic state of children on CRRT should therefore be monitored closely and frequent controls of the electrolyte concentrations should be performed.

## Key messages

• The frequency of complications in children on CRRT is high, and some of these complications can be serious.

• The most common complications are hypotension at the time of connection and electrolyte disturbances. The hemodynamic state of children on CRRT should be monitored closely and frequent controls of the electrolyte concentrations should be performed.

## Abbreviations

ALT: alanine transferase; CRRT: continuous renal replacement therapy; MBP: mean blood pressure; PELOD score: paediatric logistic organ dysfunction score; PIM score: pediatric index of mortality score; PRISM score: pediatric risk of mortality score.

## Competing interests

The authors declare that they have no competing interests.

## Authors' contributions

MJS and JLH conceived the study and participated in the design, data collection and analysis, and drafting of the manuscript. JU, MJS, YB and MB participated in the data collection and analysis of data, and drafting of the manuscript. JMB participated in the design of the study and performed the statistical analysis. All authors read and approved the final manuscript.
